# Characterizing best practices for tonsil-oral-scrubbing (TOSc) collection for PRRSV RNA detection in sows

**DOI:** 10.1186/s40813-024-00385-7

**Published:** 2024-10-07

**Authors:** Peng Li, Ana Paula Poeta Silva, Hao Tong, Paul Yeske, Laura Dalquist, Jason Kelly, Matt Finch, Amanda V. Anderson Reever, Darwin L. Reicks, Joseph F. Connor, Phillip C. Gauger, Derald J. Holtkamp, Gustavo S. Silva, Giovani Trevisan, Daniel C. L. Linhares

**Affiliations:** 1 Veterinary Diagnostic and Production Animal Medicine, Iowa State University College of Veterinary Medicine, Ames, IA USA; 2 Swine Vet Center, Saint Peter, MN USA; 3 Suidae Health and Production, Algona, IA USA; 4 Reicks Veterinary Research & Consulting, Saint Peter, MN USA; 5 Carthage Veterinary Service, Ltd, Carthage, IL USA

**Keywords:** PRRSV, Detection, Sow, TOSc, Best practice, Surveillance

## Abstract

**Background:**

A Tonsil-Oral-Scrubbing (TOSc) method was developed to sample the sow’s oropharyngeal and tonsillar area without snaring and has shown comparable porcine reproductive and respiratory syndrome virus (PRRSV) RNA detection rates with tonsil scraping in infected sows. This study investigated the effect of specific TOSc collection factors on the PRRSV RT-rtPCR results (detection rates and Ct values). Those factors include whether the sow was snared or not snared at TOSc collection (“snared” vs. “not snared”); whether the sow was laying down or standing at collection (“laying down” vs. “standing”); and type of collectors used for TOSc collection (“TOSc prototype” vs. “Spiral-headed AI catheter (SHAC)”). Volume of fluid was compared between “snared” and “not snared” groups, and collection time was compared between “laying down” and “standing” groups as well.

**Results:**

The effect for each factor was assessed in three independent studies following the same design: TOSc was collected twice from each studied sow, once with the baseline level for a factor (“not snared”, or “standing”, or “TOSc prototype”), and another time followed by the other level of the paired factor (“snared”, “laying down”, or “SHAC”, correspondingly). Results showed that “not snared” TOSc had numerically higher PRRSV RNA detection rate (60.7% vs. 52.5%, *p* = 0.11), significantly lower median Ct values (31.9 vs. 32.3, *p* < 0.01), and significantly higher volume of fluid than “snared” samples (1.8 mL vs. 1.2 mL, *p* < 0.01); “laying down” TOSc samples did not differ statistically (60.7% vs. 60.7%) in the PRRSV RNA detection rate, obtained numerically lower median Ct values (30.9 vs. 31.3, *p* = 0.19), but took 40% less collection time compared to “standing” TOSc samples; samples collected using the “TOSc prototype” had numerically higher PRRSV RNA detection rate (91.7% vs. 88.3%, *p* = 0.27) and significantly lower median Ct values (32.8 vs. 34.5, *p* < 0.01) than that from “SHAC”.

**Conclusions:**

Under the conditions of this study best practices for TOSc collection aiming higher detection rate of PRRSV RNA while minimizing time for collection were suggested to be sampling TOSc without snaring, when sows are laying down, and using a prototype TOSc collector.

## Background

Commonly used sample types such as tongue fluid (TF) [1], processing fluid (PF) [2, 3], and family oral fluid (FOF) [4, 5], for porcine reproductive and respiratory syndrome virus (PRRSV) RNA detection primarily originate from suckling piglets and risk missing PRRSV infection in sows. As sows are a major source of PRRSV to neonatal piglets [6], undetected PRRSV in the breeding herd poses a significant challenge to the success of virus control and elimination programs. Commonly used sample types to detect various pathogens in sows include serum and tonsil scraping [7, 8]. While tonsil scraping was documented as preferred sample type to detect PRRSV RNA due to localized virus genome persistence in lymphoid tissues for an extended period, i.e., up to 251 days [8], serum collection and tonsil scraping are time consuming and labor intensive for large screening purposes, especially in low PRRSV prevalence scenario(s) when large sample sizes are needed. Moreover, both methods require restraining the sows, impacting animal welfare. Thus, an easy and practical alternative to collect sow samples is needed.

We recently developed a sow sampling tool, namely tonsil oral scrubbing (TOSc) collector, adapted from a sow collector used in the test-and-removal of African swine fever virus (ASFV) infected sows in China [9]. TOSc takes biological samples consisting of fluids and cells from the oropharyngeal and tonsillar area of a sampled sow within seconds, without the necessity of snaring the sows, and showed comparable detection rate with tonsil scraping in 30 acutely infected sows [9]. However, we hypothesize that the PRRSV RNA concentration in TOSc hinges on the collection of biological material from the tonsillar area and depends upon several factors observed in the field. Those factors include whether the sow was snared or not snared at collection (“snared” vs. “not snared”); whether the sow was laying down or standing at collection (“laying down” vs. “standing”); and type of collectors used for collection (“TOSc prototype” vs. “spiral-headed AI catheter (SHAC”). Thus, the objective of this study is to assess the effects of three abovementioned paired factors on PRRSV RNA detection rate and Ct values from TOSc in sows to determine the “best practices” of TOSc collection to detect PRRSV RNA.

## Methods

The Institutional Animal Care and Use Committee (IACUC) of Iowa State University, Iowa, approved this study (IACUC-22-101). The effect of three factors on PRRSV RNA detection, sample volume and collection time by TOSc samples were evaluated separately using similar study design.

### Herd selection criteria and sample size calculation

Two breeding herds 30 days within live virus inoculation (LVI) were selected. The PRRSV status of the two herds before outbreak were status II and above [10]. Sample size was calculated to be 60 sows based on the assumption of an effect size of 15% difference in PRRSV RNA detection rate at prevalence of 90%, with alpha level of 5% and 80% power.

### LVI procedure

To perform LVI, serum was collected from suckling piglets with clinical signs of PRRS (fuzzy, weak, fevering, etc.) within 30 days of outbreak by the herd veterinarian and sent to a veterinary diagnostic laboratory for PRRSV RNA by qPCR. PRRSV RNA was confirmed positive and the samples were pooled and stored in a -20℃ freezer until use. As per veterinary recommendations, serum was diluted with PBS to appropriate CTs (around 30 for both farms). One to two mL of diluted serum was injected intramuscularly into each gilt and sow in the herd..

### Comparing the factor of “not snared” with “snared”

**Fig. 1:**
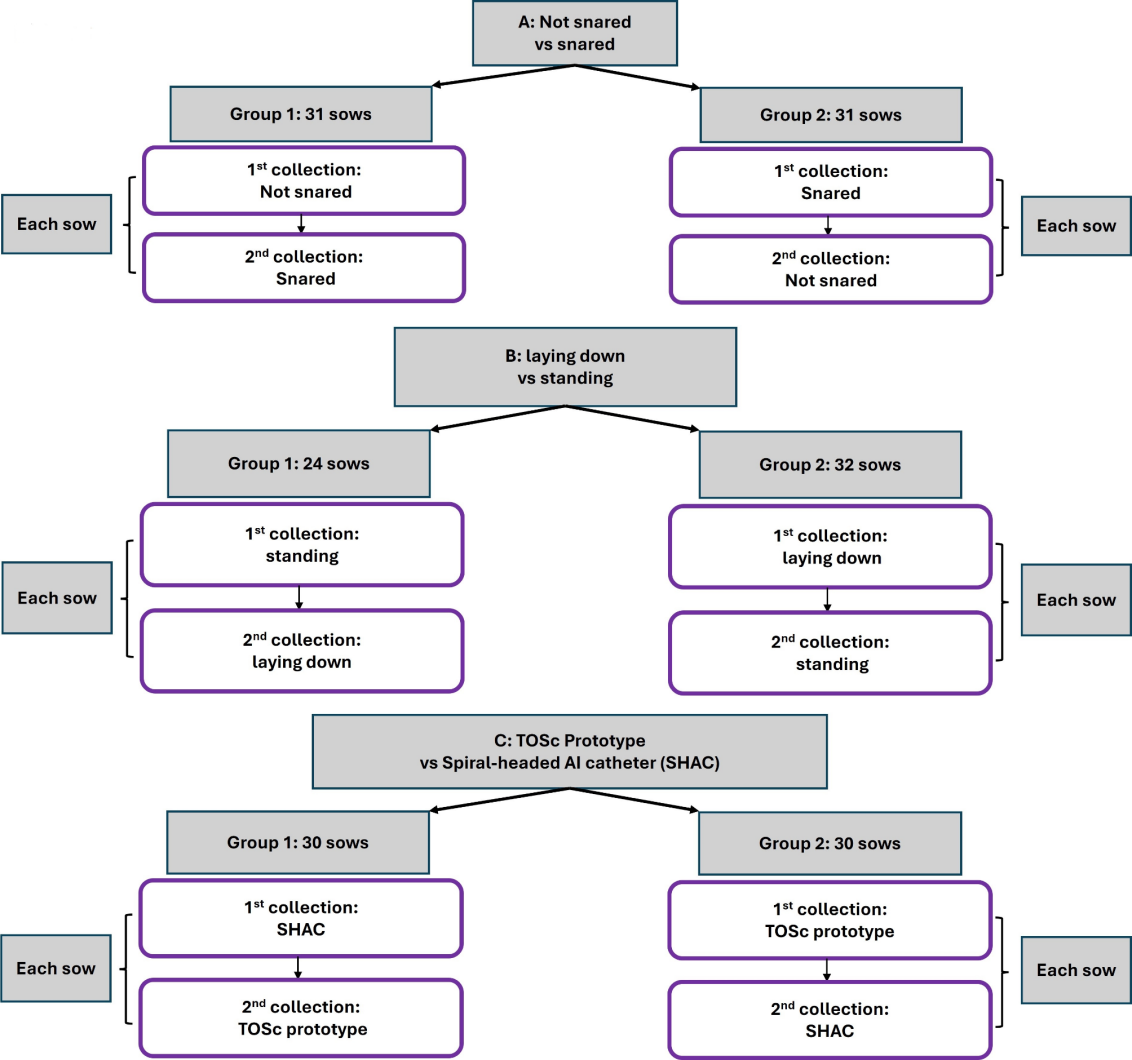
Schematic chart of study design. (**A**) To compare the factor of “not snared” with “snared”. (**B**) To compare the factor of “laying down” with “standing”. (**C**) To compare the factor of “TOSc prototype” with “SHAC”

To compare the factor of “not snared” with “snared”, 62 gestation sows were conveniently selected 30 days after LVI from breeding herd A located in Minnesota. TOSc was collected twice from each sow using the TOSc prototype collector, once when the sows were not snared, and another time when the sows were snared. The 62 animals were conveniently split into two groups with 31 sows each. For group 1, TOSc was collected first when sows were not snared, then collected again when the same sows were snared. For group 2, the collection order was reversed with first TOSc being collected when the sows were snared (Fig. 1A).

**Fig. 2:**
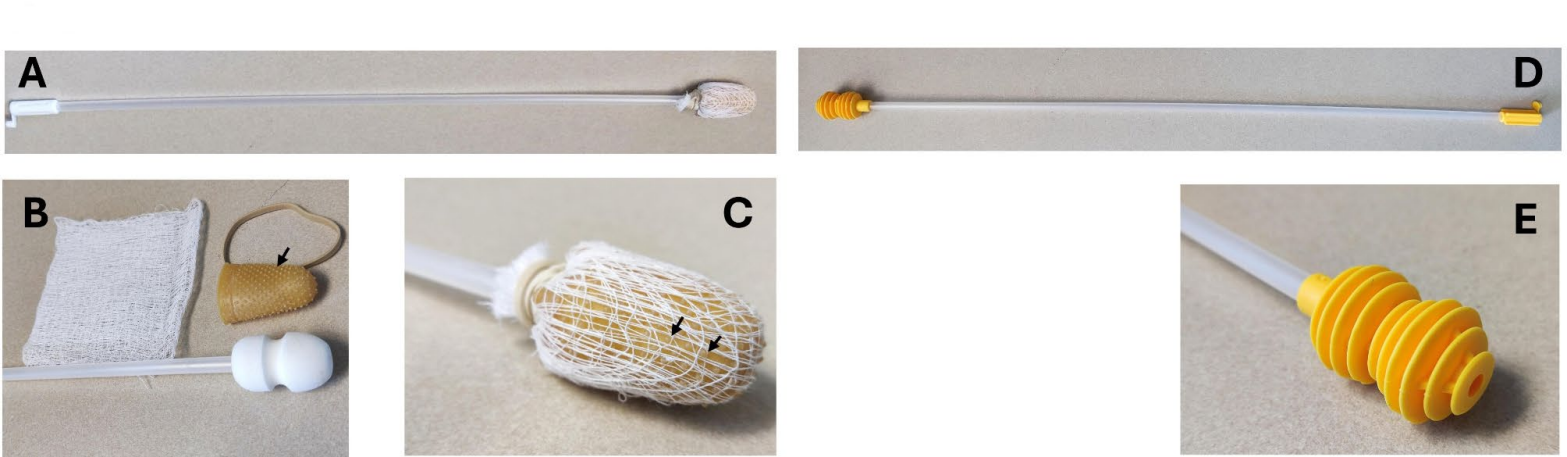
TOSc prototype and spiral-headed AI catheter (SHAC). (**A**) TOSc prototype complete picture. (**B**) Head part of TOSc prototype collector before assembly, including the head of an AI catheter, a rubber thimble, a piece of cotton gauze and a rubber band. (**C**) Head part of TOSc prototype collector after assembly. (**D**) SHAC complete picture. (**E**) Head part of SHAC. Black arrows in B and C indicated rubber spikes before and after assembly, respectively

The TOSc prototype collector was constructed using a commercially available artificial insemination (AI) rod (Golden Pig Catheter, Model 320100 from IMV International Corporation, Minneapolis, USA), a rubber thimble (Large Finger Pad, Model 098130 from Staples Inc, Framingham, USA) covered by a layer of a 10.16 cm by 10.16 cm cotton gauze (Model 074144, from Honeywell Inc, Charlotte, USA), and a rubber band to attach the cotton gauze to the AI rod (Fig. 2).

TOSc was collected without snaring the sows as previously described [9]. Briefly, the head of the collector was passed over the hard palate and pointed against the tonsillar area with an upwards angle and swept back and forth for ten seconds. The qualified sample was viscous and mucous-like. The sample was then transferred to a 50 mL falcon tube (Corning Science Mexico S.A. de C.V., Tamaulipas, Mexico), pre-deposited with 3 mL of PBS. The samples were then vortexed for 10 s and poured into a 5 mL conical tube (Corning Science Mexico S.A. de C.V., Tamaulipas, Mexico).

For TOSc samples collected when the sows were snared, the sows were restrained with a snare, and the mouths were held open with a metal mouth gag. Then the head of the collector was directed towards the tonsillar area of the mouth and moved back and forth for ten seconds and samples were transferred in the same way as TOSc was collected from sows not snared as described above. 

Volume of TOSc samples collected from “snared” and “not snared” groups were measured using a 5-mL serological pipette (Nunc serological pipette, Thermo Scientific, USA) attached to an electronic manual pipettor (EP-PRO, JOANLAB, Hangzhou, China). Briefly, each sample was suspended in the 5 mL falcon tube and drawn by the serological pipette. The volume was then read on the pipette and recorded to one decimal place.

### Comparing the factor of “laying down” with “standing”

Likewise, to compare the factor of “laying down” with “standing”, another 56 gestation sows were conveniently selected 30 days after LVI from breeding herd A. TOSc was collected twice from each of sow without snaring using TOSc prototype collector, once when the sows were standing in the morning and another time while they were laying down in the afternoon. For group 1, TOSc was collected from 24 sows first when they were standing in the morning, and then collected again when they were laying down in the afternoon. For group 2 with 32 sows, the collection order was reversed with first TOSc being collected when the sows were laying down in the afternoon and the second TOSc being collected when they were standing up the next morning (Fig. 1B).

Collection time for TOSc samples from “standing” and “laying down” groups were recorded using a digital timer (Taylor digital kitchen timer, Taylor USA, California, USA). Time recording started from approaching the sows and ended when the TOSc collection device was removed from the sow’s oral cavity. The collection time was recorded to two decimal places.

### Comparing the factor of “TOSc prototype” with “spiral-headed AI catheter (SHAC)”

The same design was implemented to compare the factor of the “TOSc prototype” with the “Spiral-headed AI catheter (SHAC)” (Fig. 1C). The SHAC is a commercially available AI catheter with a spiral shaped head (Fig. 2), increasing the contact between catheter and reproductive tract (Goldenpig V2 catheter, IMV International Corporation, Minneapolis, USA). Sixty sows were conveniently selected 2 weeks after live-virus inoculation (LVI) from the breeding herd B in Iowa. TOSc was collected twice from each sow when the sows were standing and not snared, once with the TOSc prototype collector and another time with SHAC. The 60 animals were conveniently split into two groups with 30 sows each. For group 1, TOSc was collected first with SHAC and then collected with TOSc prototype. For group 2, the collection order was reversed with first TOSc being collected with TOSc prototype (Fig. 1C).

### Diagnostic testing

All samples were tested at the Iowa State University Veterinary Diagnostic Laboratory for PRRSV RNA by RT-rtPCR using previously validated commercially available assays. Test results having cycle threshold value (Ct value) < 40 were considered PRRSV RNA-positive.

### Statistical analysis

A logistic mixed regression model was used to assess the difference in the PRRSV RNA detection rate as a function of paired factors and collection order, using sow ID as a random effect, and the Tukey-Kramer test was used to compare the post hoc pairwise differences in the detection rate between paired factors. All analyses used the package lme4 from R program 4.2.2 (R Core Team, 2019).

The signed rank test [11] was performed to assess if there was a difference in the Ct values, sample volumes, and collection time. An alpha of 0.05 was used to determine statistical significance.

## Results

### Comparison of PRRSV RNA detection rate, Ct values, and sample volume between TOSc samples from “not snared” sows and “snared” sows

**Fig. 3:**
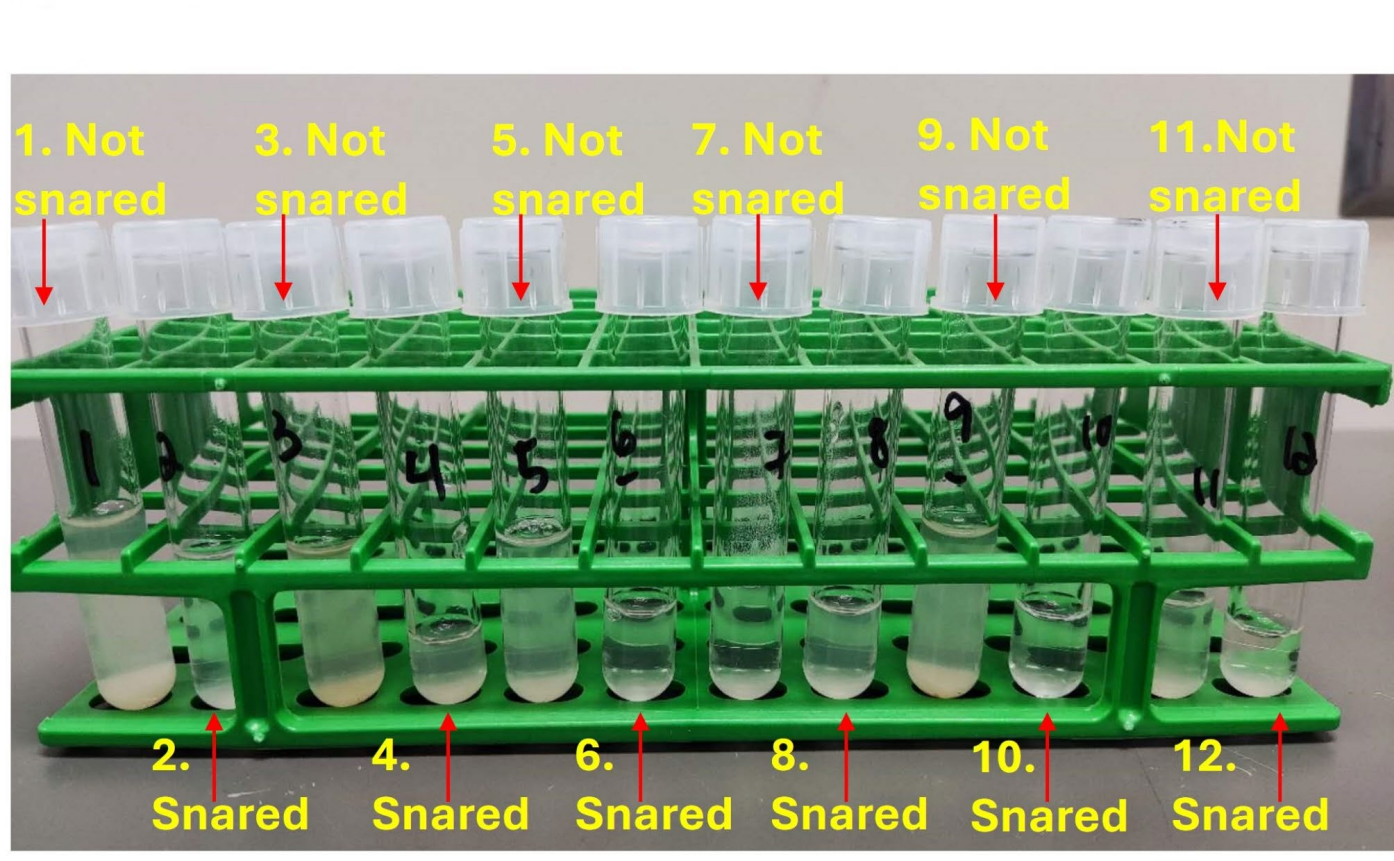
Picture of TOSc samples from “not snared” and “snared” groups. Odd numbers (1, 3, 5, 7, 9, 11) were from sows that were not snared. Even numbers (2, 4, 6, 8, 10, 12) were from snared sows. Consecutive numbers (1 and 2, 3 and 4, until 11 and 12) were from the same sow

TOSc samples from “not snared” sows showed numerically but not statistically (*p* = 0.11, Tukey’s test) higher PRRSV RNA detection rate (60.7%; 95% confidence interval (CI), 48.0–72.0%) than samples from “snared” sows (52.5%; 95% CI, 40.0-64.6%). Likewise, the effect of collection order was not statically significant (*p* = 0.76, logistic mixed regression). TOSc samples from “not snared” sows exhibited significantly lower (*p* < 0.01, signed rank test) median Ct values (31.9) than TOSc samples from “snared sows” (32.3). Significantly higher (*p* < 0.01, signed rank test) median liquid volume were also observed in TOSc samples from “not snared” sows (1.8 mL, 95% CI, 1.3-2.5mL) than that from “snared” sows (1.2 mL, 95% CI, 0.4–2.1 mL). (Table 1). Visually, TOSc samples from “not snared” sows were opaquer and yielded more deposits than that from “snared” sows (Fig. 3).

**Table 1:** Comparison of PRRSV RNA detection rate, ct values, and sample volume between TOSc samples from “not snared” and “snared” sows

	Not snared	Snared
PRRSV RNA detection rate (95% CI)	60.7% (48.0–72.0%)^**a**^	52.5% (40.0-64.6%)^**a**^
Median Ct values (lowest-highest)	31.9 (26.4–38.1)^**a**^	32.3 (27.7–39.1)^**b**^
Median sample volume (lowest-highest) /mL	1.8 (1.3–2.5)^**a**^	1.2 (0.4–2.1)^**b**^

Different letters “a, b” indicate significant differences in the median Ct values or sample volume by signed ranked test. Same letter “a” in row “PRRSV RNA detection rate (95% CI)” indicate no significant difference in least-square means of PRRSV detection rate between “not snared” and “snared” groups by Tukey’s test

### Comparison of PRRSV RNA detection rate, Ct values, and collection time between TOSc samples from “laying down” sows and “standing” sows

TOSc samples from “laying down” sows showed no statistical different PRRSV RNA detection rate (60.7%; 95% CI, 48.0–72.0%) compared with samples from “standing” sows (60.7%; 95% CI, 48.0–72.0%). The order of the collection was not statistically significant (*p* = 1.0, logistic mixed regression). TOSc samples from “laying down” sows exhibited numerically lower median Ct values (30.9) than those from “standing” (31.3) sows, but the difference was not significant (*p* = 0.19, signed rank test). The median time was significantly lower (*p* < 0.01, signed rank test) to collect TOSc samples from sows that were “laying down” (15.4 s) than sows that were “standing” (25.4 s) (Table 2).

**Table 2:** Comparison of PRRSV RNA detection rate, ct values, and collection time between TOSc samples from “laying down” and “standing” sows

	Standing	Laying down
PRRSV RNA detection rate (95% CI)	60.7% (48.0–72.0%)^**a**^	60.7% (48.0–72.0%)^**a**^
Median Ct values (lowest-highest)	31.3 (25.9–37.9)^**a**^	30.9 (25.1–39.1)^**a**^
Collection time/ seconds	25.4 (11.0-159.8)^**a**^	15.4 (8.6–37.1)^**b**^

Different letters “a, b” indicate significant differences in the median collection time by signed ranked test. Same letter “a” in row “PRRSV RNA detection rate (95% CI)” indicate no significant difference in least-square means of PRRSV detection rate between “standing” and “laying down” groups by Tukey’s test. Same letter “a” in row “median Ct values” indicate no significant difference in median Ct values between “standing” and “laying down” groups by signed rank test

### Comparison of PRRSV RNA detection rate and Ct values between TOSc samples collected with “TOSc prototype” and “SHAC”

TOSc samples collected using “*TOSc prototype*” showed numerically higher PRRSV RNA detection rate(91.7%; 95% CI, 79.5-96.9%) than that collected using “SHAC” (88.3%; 95% CI, 75.5-94.9%). The difference was not statistically significant (*p* = 0.27, Tukey’s test). The effect of collection order was not statistically significant (*p* = 0.84, logistic mixed regression). TOSc samples collected from “TOSc prototype” exhibited significantly lower (*p* < 0.01, signed rank test) median Ct values (32.8) compared to collection with the “SHAC” (34.5) (Table 3).

**Table 3:** Comparison of PRRSV RNA detection rate and ct values between TOSc samples collected using “TOSc prototype” and that using “SHAC”

	TOSc prototype	SHAC
PRRSV RNA detection rate (95% CI)	91.7% (79.5–96.9%) ^a^	88.3% (75.5–94.9%) ^a^
Median Ct values (lowest-highest)	32.8(28.2–37.4) ^a^	34.5(28.6–38.9) ^b^

Different letters “a, b” indicate significant differences in the median Ct values by signed ranked test. Same letter “a” in row “PRRSV RNA detection rate (95% CI)” indicate no significant difference in least-square means of PRRSV detection rate between “TOSc prototype” and “SHAC” groups by Tukey’s test. SHAC: spiral-headed AI catheter

## Discussion

TOSc targets the caudal portion of the oral cavity, including the soft palatine tonsils, which are the reference location to detect PRRSV RNA due to the extended and localized virus genome presence in lymphoid tissues [8]. Thus, collection procedures or collectors influencing the collection of biological samples from the soft palatine tonsillar area will most likely affect TOSc performance to detect PRRSV RNA. Here we demonstrated three factors having effects on PRRSV RNA detection by TOSc samples.

First, TOSc collected from sows that were not snared showed numerically higher PRRSV RNA detection rate and significantly lower median Ct values than that from snared sows. This may be due to TOSc collection when sows were not snared activated the chewing and swallowing reflex and increased the frequency and pressure of contact between the TOSc collector and tonsillar area. In the field, we observed that TOSc collector was pressed to the soft palatine tonsil by the tongue after swallow reflex activation. On the contrary, when the sow was snared and mouth held open, it was less likely that TOSc collector had firm contact with the soft palatine tonsil area to scrub off biological materials. This was consistent with the finding in this study that more volume of fluid were observed from TOSc samples collected from “not snared” sows compared with “snared” sows. In this study, we increased the volume of pre-deposited PBS in the falcon tube to 3 mL from 2 mL as previously described [9]. That’s because when we pre-deposited the tube with 2 mL PBS, even rare, it was possible that we would get less than 0.5 mL fluid. The standard veterinary diagnostic laboratory practice requires to submit at least 0.5 mL fluid for extraction, otherwise additional PBS will be added for this individual sample, which will result in inconsistency to compare among samples. In this sense, we changed the volume of PBS to 3 mL. TOSc can only be collected without snaring when the sows are in gestation stalls or farrowing crates, where their movement is limited compared to open pens. When the gilts and sows are penned in groups, snaring is required for TOSc sampling. Thus, the effect of snaring on TOSc collection to detect PRRSV RNA has practical field implications. When TOSc are collected from snared sows or gilts housed in group pens, the field practitioners should be aware that PRRSV RNA detection may be compromised and ultimately affect the ability to make informed decisions.

Second, TOSc samples from “laying down” sows yielded identical PRRSV RNA detection rate and numerically lower median Ct values compared with TOSc samples from “standing” sows. In contrast, the collection time was significantly lower in “laying down” sow groups compared with “standing” groups, with a 40% reduction in median values from 25.4 s to 15.4 s. Moreover, extreme values (159.8 s) were present in “standing” groups, while the maximum collection time was 37.1 s in “laying down” groups. In this study, we failed to collect planned numbers of sows in the first group in which TOSc collection while sows were standing preceded TOSc collection while they were laying down because quite a few already laying down sows stood up when we approached them for the second sampling. So those sows had to excluded from the study. On the contrary, the second group did not have the same problem of not getting planned sample size because we could always get the laying down sows to stand up for the second TOSc collection. As was observed in the field, when sows were standing, for example, in the morning, they were more active and prone to dodge collection, giving rise to extended collection time and more difficulties for collecting biological samples from the tonsillar area. On the contrary, the TOSc collection seemed much easier when the sows were laying down and more cooperative with the sampling process. The 40% reduction of collection time will have important applications for field veterinarians and practitioners, especially when collecting large numbers of TOSc samples in low prevalence scenarios. Test and removal of positive sows based on serological results using serum samples has been reported as a method for eliminating PRRSV in sow farms. However, the reported herd sizes were small relative to the USA’s current average, ranging from 200 to 1500 sows [12]. As the field practitioners are dealing with much larger herd sizes nowadays, using molecular assays on TOSc samples, a more labor efficient and animal friendly sample type than serum, ,to test and remove PRRSV-positive sows at the end of herd closure might serve as a potentially practical approach for achieving stability sooner.

Thirdly, TOSc samples collected with TOSc prototype collector showed numerically higher PRRSV RNA detection rate and significantly lower median Ct values than SHAC. The SHAC was chosen as an analogue of TOSc prototype because it has a spiral-shape head, which is believed to increase the contact between collector and tonsillar area. However, the TOSc prototype collector incorporates a specific rubber thimble design (Fig. 2), which has rubber spikes making the collector more abrasive when contacting the tonsillar area. This is consistent with our definition of TOSc as a “scrubbing” process which utilizes a rubber thimble to allow a longer and more aggressive contact to “scrub off” lymphoid tissues from the caudal portion of the oral cavity including soft palatine area compared to standard swabbing methods [13, 14]. This specific rubber thimble design also makes TOSc different from oropharyngeal swabs using an artificial insemination catheter to collect samples in the test and removal of ASFV infected sows in China [9, 15]. While the TOSc prototype collector takes around 1 min to assemble and costs an additional USD 0.20 for each rubber thimble, the SHAC is a ready-to-use tool. Swine practitioners can make informed decisions based on this study about which tool to choose according to their needs in the field.

The major outcomes for this study were PRRSV RNA detection rate and Ct values, with sample volume and collection time being secondary outcomes, which were not collected for every set of factors. Besides the major factors, we also explored the interaction between collection order and detection rate based on the assumption that first collection might scrub off the epithelium cells and expose the lymphoid partition of soft palatine tonsil and increase the detection rate for second collection. But results showed that collection order did not have a significant effect on PRRSV RNA detection rate in all three studies, rejecting the null hypothesis. Results from this study can be extrapolated to farms sharing similar characteristics to the ones from this study, including crate gestation and PRRSV high prevalence. One limitation of this study was that all samples were collected from high prevalence farms, which might explain the numerically higher but not significantly different detection rate in “not snared” and “TOSc prototype” groups compared with “snared” and “SHAC” groups, respectively, based on a sample size of 60 animals. The other limitation was that the three paired factors were assessed separately, further studies will be needed to evaluate the interactions among those factors.

## Conclusions

Under the conditions of this study best practices for TOSc collection aiming higher detection rate of PRRSV RNA while minimizing time for collection were suggested to be sampling TOSc without snaring, when sows are laying down, and using a prototype TOSc collector.

## Data Availability

No datasets were generated or analysed during the current study.
